# A video protocol for rapid dissection of mouse dorsal root ganglia from defined spinal levels

**DOI:** 10.1186/s13104-020-05147-6

**Published:** 2020-06-24

**Authors:** James N. Sleigh, Steven J. West, Giampietro Schiavo

**Affiliations:** 1 Department of Neuromuscular Diseases, UCL Queen Square Institute of Neurology, University College London, London, WC1N 3BG UK; 2 UK Dementia Research Institute, University College London, London, WC1E 6BT UK; 3 Sainsbury Wellcome Centre, University College London, London, W1T 4JG UK; 4 Discoveries Centre for Regenerative and Precision Medicine, University College London Campus, London, WC1N 3BG UK

**Keywords:** Afferent nerve, Charcot-Marie-Tooth disease (CMT), Comparative anatomy, Neuroanatomy, Nociception, Pain, Peripheral nerve, Peripheral neuropathy, Sensory neuron, Spinal cord

## Abstract

**Objective:**

Dorsal root ganglia (DRG) are heterogeneous assemblies of assorted sensory neuron cell bodies found in bilateral pairs at every level of the spinal column. Pseudounipolar afferent neurons convert external stimuli from the environment into electrical signals that are retrogradely transmitted to the spinal cord dorsal horn. To do this, they extend single axons from their DRG-resident somas that then bifurcate and project both centrally and distally. DRG can be dissected from mice at embryonic stages and any age post-natally, and have been extensively used to study sensory neuron development and function, response to injury, and pathological processes in acquired and genetic diseases. We have previously published a step-by-step dissection method for the rapid isolation of post-natal mouse DRG. Here, the objective is to extend the protocol by providing training videos that showcase the dissection in fine detail and permit the extraction of ganglia from defined spinal levels.

**Results:**

By following this method, the reader will be able to swiftly and accurately isolate specific lumbar, thoracic, and cervical DRG from mice. Dissected ganglia can then be used for RNA/protein analyses, subjected to immunohistochemical examination, and cultured as explants or dissociated primary neurons, for in-depth investigations of sensory neuron biology.

## Introduction

Dorsal root ganglia (DRG) are bilateral assemblies of sensory neuron somas, satellite cells, fibroblasts and capillaries, found within dorsal intervertebral foramina at every level of the spinal column [1]. Sensory neurons possess a single axon that splits into two major branches; one projects from the DRG to connect with the spinal cord dorsal horn, while the other branch, which ends in one of many different specialised sensory receptors [2], targets peripheral tissues (e.g. skin and muscle) to detect external stimuli [3]. Neurons found within DRG can be divided into broad functional classes, e.g. nociceptors for sensing noxious stimuli and mechanoreceptors for sensing touch [4]. These can be further subcategorised based on shared transcriptomic profiles [5, 6], which emerge post-natally in response to target tissue-derived factors (e.g. neurotrophins) [7]. DRG have been isolated from several different mammalian species [8–12], and even human organ donors [13], and studied to better understand sensory neuron biology, including responses to nerve injury and disease [14–16], with experiments on mouse ganglia being most common. Mice have 30 or 31 DRG pairs, depending on genetic background [17–19]: eight cervical (C1 to C8), 13 thoracic (T1 to T13), five or six lumbar (L1 to L5/L6) and four sacral (S1 to S4). Lumbar DRG are most often studied; however, ganglia at different spinal levels show clear distinctions not only in development, size, cell number and proportion of functional subtypes, but also in their disease involvement [20–22]. It is therefore important to examine DRG from several distinct and defined spinal levels.

We have previously published a stepwise protocol for the rapid isolation of mouse DRG [9]. Here, we now extend this method with videos detailing all steps of the dissection process, as well as provide clear instruction on how to extract ganglia from defined cervical to lumbar spinal levels.

## Main Text

### Materials and methods

#### General

**Fig. 1:**
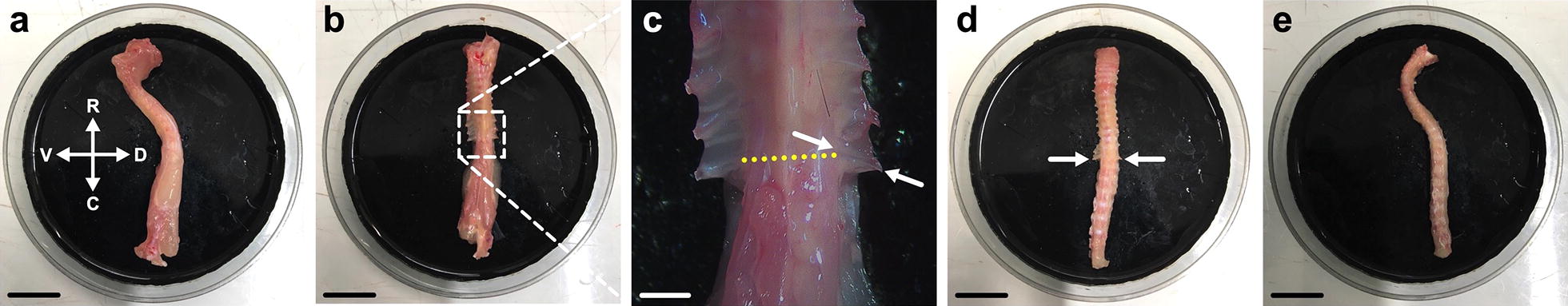
Spinal column isolation and cleaning for bisection. **a** Extract the spinal column from the base of the skull to just caudal to the femurs, as outlined previously [9]. A side aspect of the column is presented. R, rostral; C, caudal, D, dorsal; V, ventral. **b, c** Using curved scissors, carefully cut away tissue (e.g. muscle) surrounding the column so that the most caudal pair of ribs, which are floating ribs, can be identified (arrows in c identify each end of a single rib from this pair, and the dashed line indicates exactly where to transect the column in a later step of the procedure). The ventral aspect of the column is presented in both panels. Panel c is the magnified region outlined by the dashed box in b. **d** To aid subsequent bisection down the mid-line, remove additional tissue exterior to the column, leaving intact the most caudal pair of ribs (arrows) to locate the spinal level. The ventral aspect of the column is presented. (**e**) The same side aspect is presented as that shown in **a**, highlighting the impact of tissue removal. See Additional file 1 for a video depicting the subsequent steps of spinal column transection and bisection. Scale bars = 1 cm (**a**, **b**, **d**, **e**) and 1.5 mm (**c**)

The animals used in this protocol were 28 day-old female C57BL6/J mice, which were transcardially perfused with phosphate buffered saline (PBS) to decrease blood contamination (optional step). Mice can also be perfusion-fixed if taking DRG for immunofluorescence analysis. The dissection can be performed from about post-natal day 5 (P5) onwards, with alternative options available for embryonic DRG [23, 24]. All images and videos (Additional files 2, 3, 4) were taken using a DSK 500 dual head stereo microscope (Motic, Barcelona, Spain, PM5539B901) with attached Moticam 1080 HDMI digital camera (Motic, MC1080), except for Fig. 1a, b, d, and e, and video 1 (Additional file 1), which were recorded using an iPhone 6 s (Apple, Cupertino, CA). To preserve neuronal health for downstream applications, all steps should be completed efficiently and tissue kept as cold as possible. Use of a laminar flow hood will reduce contamination frequency of primary cultures [19], but is not strictly necessary.

#### Reagents, equipment and set-up

All equipment and reagents have been described previously [9].

#### Dissection

While we have described mouse DRG dissection in a previous protocol [9], we did not provide sufficient direction for accurate isolation of ganglia across specific spinal levels. Here, we will thus explain the process from spinal column excision to DRG removal from defined lower lumbar to upper cervical spinal levels, with reference to videos (Additional files 1, 2, 3, 4). It is important to note that we depict DRG dissection from a C57BL6/J mouse, which usually possesses six lumbar spinal levels [17].

##### Spinal column excision

Spinal column excision can be performed in any manner that results in its isolation from the base of the skull to beyond the femurs, while leaving the most caudal pair of ribs intact. We use the following method, which is illustrated in the original protocol [9]. Cull the animal and confirm death using an appropriate method approved by your local ethics committee. Lightly wet the fur with ethanol to restrict the spread of hair. Remove the pelt by grasping the skin at the back of the animal, making an incision in the region of the lower back/femurs, and cutting/tearing the skin around the circumference of the animal in the transverse plane. Pull the pelt up towards the arms/head and cut through the skin up and along the mid-line to reveal the skull. Cut away the arms beneath the scapulas and remove the head by cutting as close to the base of the skull as possible. Turn the mouse over and make an incision through the ventral abdominal wall that covers the intestines. Cut laterally along the musculature to the spinal column, turn the scissors 90˚ so that the blades are parallel with and adjacent to the column, and cut up towards the head, through the 13 pairs of ribs [18], until the tip of the scissor blade becomes visible. Be careful to not cut away all of the most caudal rib, as this serves as a key marker in subsequent steps. Repeat on the other side of the body and then cut away the viscera ventrally attached to the column. Turn the mouse 180˚ so that the scissor blades are once again parallel and adjacent with the column and cut through both femurs. Cut and relieve the column just caudal to where the femurs were attached (Fig. 1a, b). Using curved scissors with the tips pointing away from the column, carefully remove surrounding tissues (e.g. muscle) and remaining ribs, except for the most caudal pair (Fig. 1c, d). Once thoroughly cleared, it will be easier to identify individual vertebrae and discs (Fig. 1e), which will considerably aid bisection.

##### Spinal column bisection

**Fig. 2:**
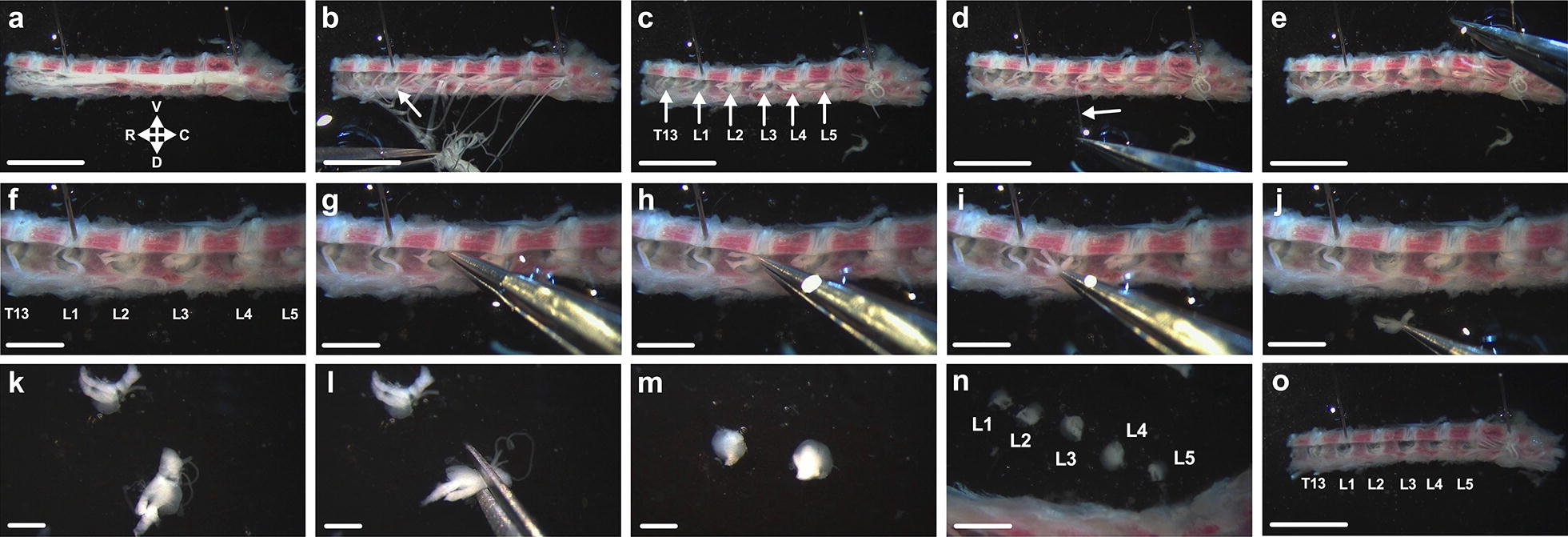
Lumbar DRG extraction. **a** Using insect pins placed through two of the soft, white vertebral discs towards each end of the hemi-segment, affix the lumbar spinal column to the Sylgard dish with the spinal canal facing outwards. R, rostral; C, caudal, D, dorsal; V, ventral (orientation maintained in all panels). **b** Remove the bisected spinal cord. Note that the centrally projecting axons emanating from DRG can be seen and used to trace the location of the ganglia (arrow identifies lumbar level 1 [L1] DRG). **c** DRG reside within dorsal intervertebral foramina in the spinal canal, level with each disc (arrows). When the column is cut in half at the most caudal rib pair (see Additional file 1), the thoracic level 13 (T13) DRG is the first ganglion found at the rostral end of the lower column half, followed by L1 to L5. **d, e** Remove the meninges that line the spinal canal, covering the DRG. This is best done starting at one end and peeling back towards the other (left to right, here). The arrow highlights the membrane clasped by forceps. **f** T13 to L5 DRG are easily visible in situ. **g–j** Pick up individual DRG using their distally projecting (*i.e.* towards the dish) axon bundle, found within each foramen (L2 DRG removed, here). Pay attention to avoid touching the DRG directly. **k–m** Identify and remove axons from extracted DRG. **n** Lumbar DRG increase in size from L1 to L4. L4 is considerably larger than L5, as are the associated axon bundles. Identification of L4 DRG is a useful way to confirm spinal level. **o** Once all required ganglia have been removed, the column can be discarded. For videos of steps a-j and k–o see Additional files 2 and 3, respectively. Scale bars = 5 (a**–**e, o), 2 (f**–**j, n) and 1 (k**–**m) mm

**Fig. 3:**
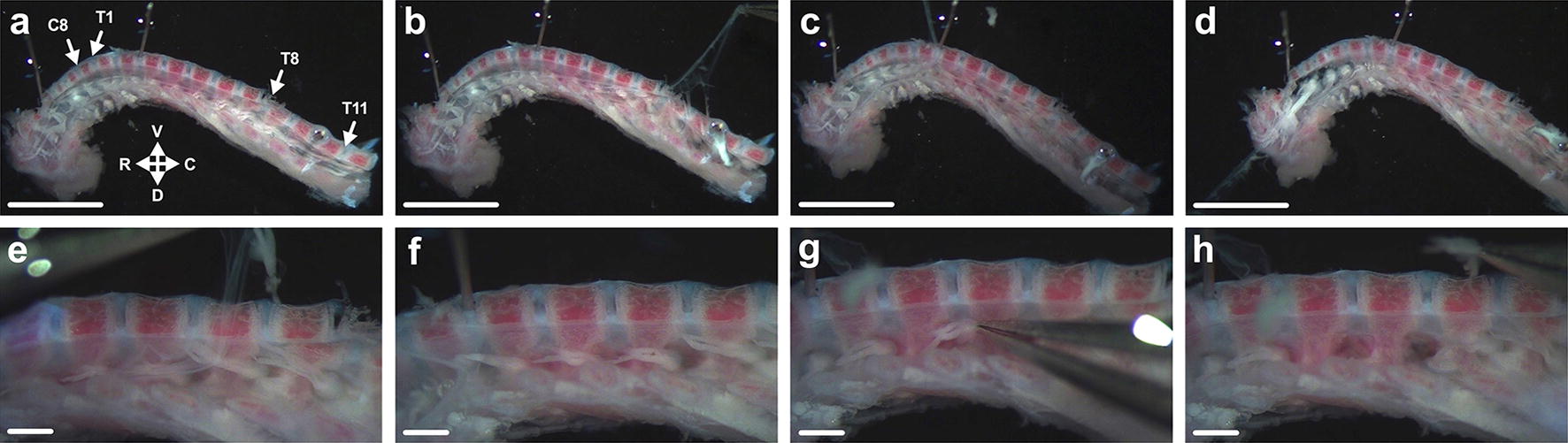
Thoracic to cervical DRG extraction. **a** Affix the spinal column segment containing thoracic and cervical levels to the Sylgard plate using three insect pins placed through vertebral discs—one each in the approximate regions of T10-T11, T2-T3, and cervical level 3**–**4 (C3**–**C4). R, rostral; C, caudal, D, dorsal; V, ventral (maintained in all panels). **b–d** Remove the first membrane lining the spinal canal by clasping it in the lower thoracic region and pulling up towards the cervical level (right to left, here). Take care that cervical DRG are not removed with the membrane. **e, f** Extract a second layer in a similar fashion. DRG in the cervical region nearly always come away with this membrane, which can be useful for extracting DRG as they are individually pulled from their foramina. See Additional file 4 for how this is done. **g, h** For thoracic ganglia that remain within foramina, which is most, if not all, pick up individual ganglia from their distally projecting axon bundle as done at the lumbar level (T9 DRG removed, here). All depicted steps are shown in Additional file 4. Scale bars = 5 (a**–**d) and 1 (e–h) mm

This part of the dissection is depicted in Additional file 1. Using a surgical scalpel blade (e.g. No.22, Swann-Morton, Sheffield, UK) either with or without a handle, make a transverse cut through the dorsal aspect of the column at the most caudal ribs (see dashed line in Fig. 1c). The spinal cord can be seen through the vertebrae on the dorsal side, but not the ventral, where the bones are thicker (*i.e.* the vertebral bodies, which are clearly seen in Figs. 2 and 3). The resulting caudal and rostral segments contain thoracic level 13 (T13) to lumbar level 5/6 (L5/L6) (and beyond) and cervical level 1 (C1) to T12 DRG, respectively. With thick forceps, firmly hold the caudal segment, dorsal-side up, along its length. Using the same curved scalpel blade as above to allow a rolling motion, bisect the spinal column from one end to the other, down the mid-line creating two hemi-segments. When cutting, be careful to not let the segment slip/roll, as this will result in a more difficult DRG extraction and may damage/destroy the ganglia. Repeat this process with the rostral segment, using curved forceps to hold the rostral end.

##### Lumbar DRG extraction

This part of the dissection is depicted in Additional file 2. Move the hemi-segments to a Sylgard-lined petri dish containing ice-cooled PBS. Secure a caudal hemi-segment with the spinal canal facing outwards by putting two insect pins through intervertebral discs, one towards each end of the column (Fig. 2a). Remove the spinal cord and, by blunt dissection with forceps, cut through axon bundles that project from the in situ DRG up towards the dislodged spinal cord (Fig. 2b). Individual DRG can be observed within intervertebral foramina at the level of each disc. To orientate the segment, vertebrae become larger towards the tail end, while L3 to L5 DRG tend to be coated in dark melanin patches in C57BL6/J mice. The first DRG at the rostral end is T13, progressing caudally to L1 and onto L5 (Fig. 2c). L4 DRG are the largest, with axon bundles to match, and can be used to confirm spinal level (Fig. 2n). Starting at the rostral end, grasp the meninges, which line the spinal canal and look like translucent sheets (Additional file 5: Figure S1a), and pull back towards the caudal end (Fig. 2d, e). If they prove difficult to identify, grasp with forceps at vertebrae between the ganglia. Removal of this membranous layer, aids DRG extraction. Be careful that DRG do not get dislodged at this point. If they do, take note of the spinal level, cut and collect the meninges containing the DRG, and attempt to remove them from the membrane (pinning to the Sylgard can help). Identify the spinal level (Fig. 2f) and extract individual DRG by grasping at distal axon bundles, found within the foramina, and lifting the ganglia up and out (Fig. 2g-j). Take care not to grasp the DRG directly as this can cause damage. All DRG within a segment tend to lie in a similar orientation/angle; in both the segment imaged (Fig. 2) and video recorded (Additional file 2), DRG fall from right to left. Collect the DRG from the side with easiest access to the axons, *i.e.* from the right in these examples.

##### Lumbar DRG cleaning

This part of the dissection is depicted in Additional file 3. DRG can be removed and cleaned individually, or in groups, taking care with the second option to not confuse spinal levels. Detach the DRG axon bundles using micro spring scissors (Fig. 2k–m). To get a close cut and thus remove as much axon as possible, it can help to maneuver an axon bundle using forceps through open scissors until the DRG is adjacent to the blades before cutting. Repeat on all bundles. Sometimes, meninges remain attached to DRG after extraction (Additional file 5: Figure S1b); carefully drag or simply cut them off. T13 to L5/L6 can be isolated from this segment of the column (Fig. 2n, o).

##### Thoracic and cervical DRG extraction

This part of the dissection is depicted in Additional file 4. Using 3–4 insect pins through intervertebral discs, secure the caudal segment to the Sylgard (Fig. 3a). The most caudal DRG, found at the straight end of this hemi-segment, is T12; be aware that sometimes the transverse cut made at the caudal ribs can damage this DRG pair. To confirm spinal levels, place rostral and caudal segments next to each other and visually assess the region of the transverse cut. Remove the spinal cord and axotomise the centrally projecting bundles as done for the lumbar segment. The meninges here tend to come away in two membranous layers. Extract the first by grasping it in the lower thoracic region and pulling up towards the head end (Fig. 3b–d), once again taking care not to dislodge ganglia. Repeat this for the second layer (Fig. 3e, f). Be aware that as you reach the cervical region, the DRG will nearly always be pulled from their foramina. Use this to your advantage by collecting the DRG via their centrally or distally projecting axons with a second pair of forceps. Cervical DRG can be collected before or after thoracic DRG (Fig. 3g, h) (the latter is done in Additional file 4). Detach axons as done for the DRG of the caudal segment (Fig. 2k–m and Additional file 3).

### Discussion

By following this step-by-step video-assisted protocol, you will be able to swiftly learn how to dissect mouse sensory ganglia from defined cervical, thoracic, and lumbar levels of the spinal column (> 50 DRG/animal). This protocol and associated tools can be used to isolate ganglia from mice aged P5 onwards. The resulting DRG can be subsequently used for analyses of RNA and protein, immunohistochemically investigated as sections [15] and when cleared [14], or cultured as explants and dissociated primary neurons [19, 25] for the in vitro study of live cellular processes [22]. Moreover, assessment of sensory neuron biology can be simultaneously compared and contrasted with that of the motor nervous system [23, 26–29] for detailed peripheral nerve characterisation. This is particularly useful in mouse models of Charcot-Marie-Tooth disease [30], a life-long, hereditary neurodegenerative condition impacting both motor and sensory nerves [31].

#### Limitations

- Transecting the extracted spinal column at the last pair of ribs (Additional file 1) can damage T12 or T13 DRG. If the ganglia at these levels are specifically required, the cut can be performed at a different site, or bisection of the entire intact column can be attempted.
- Bisection of the spinal column cuts and thus limits the use of the spinal cord (e.g. prevents sectioning and immunohistochemistry), which can be avoided when DRG are extracted via dorsal laminectomy [19].
- Sacral DRG are relatively difficult to isolate due to surrounding bone structure and their small size, and thus provide limited material; they are therefore not included in this protocol.

## Supplementary information

**Additional file 1.** Spinal column bisection.

**Additional file 2.** Extraction of lumbar DRG.

**Additional file 3.** Removal of axon bundles from extracted lumbar DRG.

**Additional file 4.** Extraction of thoracic and cervical DRG.

**Additional file 5: Figure S1.** Removal of the meninges.

## Data Availability

All data generated or analysed during this study are included in this published article and its additional files.

## Abbreviation

DRG: Dorsal root ganglia

## References

[1] Haberberger RV (2019). Human dorsal root ganglia. Front Cell Neurosci..

[2] Willis WD (2007). The somatosensory system, with emphasis on structures important for pain. Brain Res Rev.

[3] Lallemend F, Ernfors P (2012). Molecular interactions underlying the specification of sensory neurons. Trends Neurosci.

[4] Emery EC, Ernfors P, Wood JN (2018). Dorsal root ganglion neuron types and their functional specialization. The oxford handbook of the neurobiology of pain.

[5] Usoskin D (2015). Unbiased classification of sensory neuron types by large-scale single-cell RNA sequencing. Nat Neurosci.

[6] Zheng Y (2019). Deep sequencing of somatosensory neurons reveals molecular determinants of intrinsic physiological properties. Neuron.

[7] Sharma N (2020). The emergence of transcriptional identity in somatosensory neurons. Nature.

[8] Ganchingco JRC (2019). Calcium imaging of primary canine sensory neurons: small-diameter neurons responsive to pruritogens and algogens. Brain Behav..

[9] Sleigh JN, Weir GA, Schiavo G (2016). A simple, step-by-step dissection protocol for the rapid isolation of mouse dorsal root ganglia. BMC Res Notes..

[10] Lin Y-T, Chen J-C (2018). Dorsal root ganglia isolation and primary culture to study neurotransmitter release. J Vis Exp..

[11] Fadda A (2016). Primary postnatal dorsal root ganglion culture from conventionally slaughtered calves. PLoS ONE.

[12] Sandercock DA (2019). Transcriptomics analysis of porcine caudal dorsal root ganglia in tail amputated pigs shows long-term effects on many pain-associated genes. Front Vet Sci..

[13] Valtcheva MV (2016). Surgical extraction of human dorsal root ganglia from organ donors and preparation of primary sensory neuron cultures. Nat Protoc.

[14] West SJ, Bonboire D, Bennett DL (2020). StereoMate: 3D stereological automated analysis of biological structures. BioRxiv..

[15] Sleigh JN (2017). Trk receptor signaling and sensory neuron fate are perturbed in human neuropathy caused by *Gars* mutations. Proc Natl Acad Sci USA.

[16] Rossor AM (2020). Loss of BICD2 in muscle drives motor neuron loss in a developmental form of spinal muscular atrophy. Acta Neuropathol Commun..

[17] Rigaud M (2008). Species and strain differences in rodent sciatic nerve anatomy: implications for studies of neuropathic pain. Pain.

[18] Cook MJ (1965). The anatomy of the laboratory mouse.

[19] Malin SA, Davis BM, Molliver DC (2007). Production of dissociated sensory neuron cultures and considerations for their use in studying neuronal function and plasticity. Nat Protoc.

[20] Lawson SN, Biscoe TJ (1979). Development of mouse dorsal root ganglia: an autoradiographic and quantitative study. J Neurocytol.

[21] Berg JS, Farel PB (2000). Developmental regulation of sensory neuron number and limb innervation in the mouse. Brain Res Dev Brain Res.

[22] Sleigh JN (2020). Altered sensory neuron development in CMT2D mice is site-specific and linked to increased GlyRS levels. BioRxiv..

[23] Wiese S (2010). Isolation and enrichment of embryonic mouse motoneurons from the lumbar spinal cord of individual mouse embryos. Nat Protoc.

[24] Leach MK (2011). The culture of primary motor and sensory neurons in defined media on electrospun poly-l-lactide nanofiber scaffolds. J Vis Exp..

[25] Fornaro M, Sharthiya H, Tiwari V (2018). Adult mouse DRG explant and dissociated cell models to investigate neuroplasticity and responses to environmental insults including viral infection. J Vis Exp..

[26] Sleigh JN (2014). Morphological analysis of neuromuscular junction development and degeneration in rodent lumbrical muscles. J Neurosci Methods.

[27] Jones RA (2016). NMJ-morph reveals principal components of synaptic morphology influencing structure-function relationships at the neuromuscular junction. Open Biol..

[28] Mech AM (2020). Morphological variability is greater at developing than mature mouse neuromuscular junctions. J Anat.

[29] Sleigh JN, Tosolini AP, Schiavo G (2020). In vivo imaging of anterograde and retrograde axonal transport in rodent peripheral nerves. Methods Mol Biol..

[30] Sleigh JN (2014). Neuromuscular junction maturation defects precede impaired lower motor neuron connectivity in Charcot-Marie-Tooth type 2D mice. Hum Mol Genet.

[31] Reilly MM, Murphy SM, Laurá M (2011). Charcot-Marie-Tooth disease. J Peripher Nerv Syst..

