# Supplementary material for: A video protocol for rapid dissection of mouse dorsal root ganglia from defined spinal levels
Source: BMC Res Notes. 2020 Jun 24;13:302. doi: 10.1186/s13104-020-05147-6 (PMC7313212; doi:10.1186/s13104-020-05147-6)
Supplement: Supplementary file 1 — Additional file 1. Spinal column bisection. [file 13104_2020_5147_MOESM1_ESM.docx]

**Additional file 1 (.MOV) Spinal column bisection.** This video shows the process of spinal column transection at the final pair of ribs (into caudal and rostral segments), followed by bisection of each segment down the mid-line creating a total of four hemi-segments. Initially, the first transverse cut is made on the dorsal aspect of the column with rostral end to the right and caudal to the left (0:10). Using straight forceps, the caudal segment (containing thoracic level 13 [T13] to lumbar level 5 [L5] DRG and beyond) is then bisected down the mid-line from the dorsal side (0:30 to 0:58). Before this, the ventral aspect with clearly visible vertebrae is highlighted with the scalpel (0:22 to 0:26). The rostral segment (containing thoracic and cervical DRG) is then cut in a similar manner, but this time using curved forceps to hold the most rostral part of the column (1:10 to 1:44). Care must be taken to cut the two segments as close to down the mid-line as possible. See also **Figure 1**, which can be used to discern scale. The file can be accessed at: <https://figshare.com/s/4b7f88e6b042cf5de372>. Video run time is 1:49 and there is no audio.
