# Supplementary material for: A video protocol for rapid dissection of mouse dorsal root ganglia from defined spinal levels
Source: BMC Res Notes. 2020 Jun 24;13:302. doi: 10.1186/s13104-020-05147-6 (PMC7313212; doi:10.1186/s13104-020-05147-6)
Supplement: Supplementary file 2 — Additional file 2. Extraction of lumbar DRG. [file 13104_2020_5147_MOESM2_ESM.docx]

**Additional file 2 (.AVI) Extraction of lumbar DRG.** This video details the pinning of the lumbar column hemi-segment to the Sylgard (spinal canal facing outwards from the dish) (0:04 to 0:14), spinal cord extraction (0:18 to 0:28), axotomy of centrally projecting DRG axon bundles with forceps (0:29 to 0:34), removal of the meninges (0:42 to 1:30) and, finally, sequential extraction of lumbar level 1 (L1) to L5 DRG (1:38 to 2:18). Care must be taken when removing DRG from their foraminae to avoid grasping the ganglia; instead, collect them via their roots. The rostral end of the hemi-segment is to the left and caudal to the right, while ventral side is towards the bottom edge of the field of view and dorsal side at the top. See also **Figure 2**, which can be used to discern scale. The file can be accessed at: <https://figshare.com/s/ff758fe38bc0f8b50908>. Video run time is 2:31 and there is no audio.
