# Supplementary material for: A video protocol for rapid dissection of mouse dorsal root ganglia from defined spinal levels
Source: BMC Res Notes. 2020 Jun 24;13:302. doi: 10.1186/s13104-020-05147-6 (PMC7313212; doi:10.1186/s13104-020-05147-6)
Supplement: Supplementary file 3 — Additional file 3. Removal of axon bundles from extracted lumbar DRG. [file 13104_2020_5147_MOESM3_ESM.docx]

**Additional file 3 (.AVI) Removal of axon bundles from extracted lumbar DRG.** This video depicts the sequential removal of axons from extracted lumbar level 1 (L1) (0:03 to 0:18) to L5 (1:25 to 1:35) DRG. A similar process should be performed for all dissected ganglia. See also **Figure 2**, which can be used to discern scale. The same DRG dissected in this video can also be seen in **Figure 2n**. The file can be accessed at: <https://figshare.com/s/44e02f57877a5162206d>. Video run time is 1:39 and there is no audio.
