# Supplementary material for: A video protocol for rapid dissection of mouse dorsal root ganglia from defined spinal levels
Source: BMC Res Notes. 2020 Jun 24;13:302. doi: 10.1186/s13104-020-05147-6 (PMC7313212; doi:10.1186/s13104-020-05147-6)
Supplement: Supplementary file 4 — Additional file 4. Extraction of thoracic and cervical DRG. [file 13104_2020_5147_MOESM4_ESM.docx]

**Additional file 4 (.AVI) Extraction of thoracic and cervical DRG.** This video shows how to extract DRG from thoracic and cervical levels of a spinal column hemi-segment. First, four pins are placed through relatively evenly spaced intervertebral discs to secure the column to the Sylgard, spinal canal-side outwards (0:00 to 0:25). The spinal cord is then discarded (0:25 to 0:40). The first membranous layer of meninges is removed from right to left (1:00 to 1:20), followed by the second layer covering the thoracic ganglia (1:34 to 1:46), taking care in both instances that the DRG are not dragged from their foramina with the membrane. DRG are then extracted using their roots, starting with thoracic level 12 (T12) and progressing sequentially to T1 (1:48 to 3:02). Care must be taken to avoid grasping and thereby likely damaging the DRG. The second membranous layer covering the cervical DRG is then dragged back from right to left and cervical level 8 (C8) to C4 DRG sequentially removed (3:15 to 4:08). Note how the cervical DRG are pulled from their foramina by the membrane, and how they can be successfully separated and collected using the forceps. The rostral end of the hemi-segment is to the left and caudal to the right, while ventral side is towards the top edge of the field of view and dorsal side at the bottom. See also **Figure 3**, which can be used to discern scale. The file can be accessed at: <https://figshare.com/s/3789eace03706b29c792>. Video run time is 4:16 and there is no audio.
