# Supplementary material for: A video protocol for rapid dissection of mouse dorsal root ganglia from defined spinal levels
Source: BMC Res Notes. 2020 Jun 24;13:302. doi: 10.1186/s13104-020-05147-6 (PMC7313212; doi:10.1186/s13104-020-05147-6)
Supplement: Supplementary file 5 — Additional file 5: Figure S1. Removal of the meninges. [file 13104_2020_5147_MOESM5_ESM.docx]

**
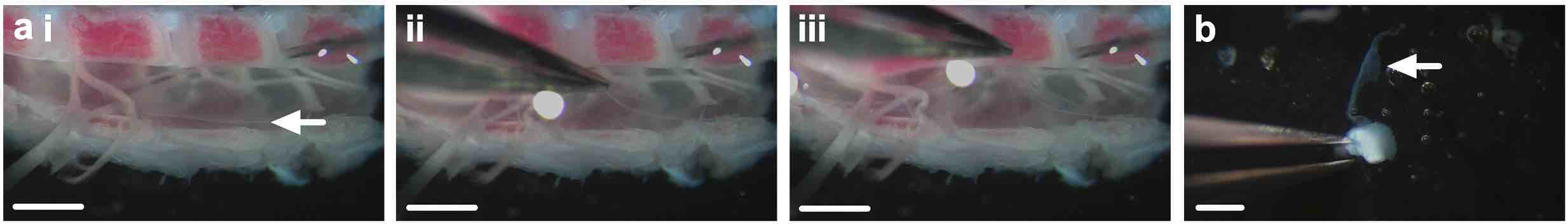
**

**Additional file 5: Figure S1. Removal of the meninges.** (**a**) Removal of the membranes that encased the spinal cord and still line the spinal canal can be challenging. Column bisection cuts the meninges in half, such that long edges of the membrane can be seen parallel with the edge of the canal (arrow in *i*). Grasp this membrane and pull to remove (*ii*, *iii*). (**b**) Meninges are often found still attached to DRG (arrow). Carefully pull these from the DRG. If this starts to damage the ganglion, cut away instead. Scale bars = 2 (a) and 1 (b) mm.
